# *C9orf72*-Derived Proline:Arginine Poly-Dipeptides Modulate Cytoskeleton and Mechanical Stress Response

**DOI:** 10.3389/fcell.2022.750829

**Published:** 2022-03-23

**Authors:** Tomo Shiota, Riko Nagata, Sotaro Kikuchi, Hitoki Nanaura, Masaya Matsubayashi, Mari Nakanishi, Shinko Kobashigawa, Noriyoshi Isozumi, Takao Kiriyama, Kazuaki Nagayama, Kazuma Sugie, Yoshito Yamashiro, Eiichiro Mori

**Affiliations:** ^1^ Department of Neurology, Nara Medical University, Kashihara, Japan; ^2^ Department of Future Basic Medicine, Nara Medical University, Kashihara, Japan; ^3^ Micro-Nano Biomechanics Laboratory, Department of Mechanical Systems Engineering, Ibaraki University, Hitachi, Japan; ^4^ Life Science Center for Survival Dynamics, Tsukuba Advanced Research Alliance, Tsukuba, Japan; ^5^ V-iCliniX Laboratory, Nara Medical University, Kashihara, Japan

**Keywords:** *C9orf72*, PR poly-dipeptides, cytoskeleton, Focal Adhesion (FA), actin

## Abstract

Proline:arginine (PR) poly-dipeptides from the GGGGCC repeat expansion in *C9orf72* have cytotoxicity and bind intermediate filaments (IFs). However, it remains unknown how PR poly-dipeptides affect cytoskeletal organization and focal adhesion (FA) formation. Here, we show that changes to the cytoskeleton and FA by PR poly-dipeptides result in the alteration of cell stiffness and mechanical stress response. PR poly-dipeptides increased the junctions and branches of the IF network and increased cell stiffness. They also changed the distribution of actin filaments and increased the size of FA and intracellular calcium concentration. PR poly-dipeptides or an inhibitor of IF organization prevented cell detachment. Furthermore, PR poly-dipeptides induced upregulation of mechanical stress response factors and led to a maladaptive response to cyclic stretch. These results suggest that the effects of PR poly-dipeptides on mechanical properties and mechanical stress response may serve as a pathogenesis of *C9orf72*-related neurodegeneration.

## Introduction

A hexanucleotide repeat expansion in *C9orf72* is common in familial amyotrophic lateral sclerosis (ALS) with frontotemporal dementia (FTD) (C9-ALS/FTD) (DeJesus-Hernandez et al., 2011), which leads to the production of proline:arginine (PR), glycine:arginine (GR), glycine:alanine (GA), proline:alanine (PA), and glycine:proline (GP) poly-dipeptides (Ash et al., 2013; Kwon et al., 2014). Arginine-rich poly-dipeptides show cytotoxicity (Kwon et al., 2014; Jovičić et al., 2015; Hao et al., 2019) and cause cell deformation and degeneration (Kwon et al., 2014; Mizielinska et al., 2014), and intermediate filaments (IFs) were found to be the binding targets of arginine-rich poly-dipeptides (Lee et al., 2016; Lin et al., 2016).

IFs regulate the focal adhesion (FA) formation and cellular mechanical properties (Kim et al., 2016; Hu et al., 2019; Laly et al., 2021). FA is responsible for adhesion to the extracellular matrix, which is formed by protein complexes such as integrin, vinculin, paxillin, focal adhesion kinase (FAK), talin, zyxin, α-Actinin, vasodilator-stimulated phosphoprotein (VASP) (Abercrombie and Dunn, 1975). Cytoskeleton and FA convert intracellular and extracellular mechanical forces into intracellular signals that induce mechanical stress responses (Hurtley, 1998; Fletcher and Mullins, 2010; López-Colomé et al., 2017). The cytoskeletal dysfunction was found as a feature of neurodegenerative diseases, for example Parkinson’s disease (McLean et al., 2000; Sweers et al., 2011; Häbig et al., 2013; Lu et al., 2017), Alzheimer’s disease (Bamburg and Bloom, 2009; Levy Nogueira et al., 2016), and ALS (Wu et al., 2012; Smith et al., 2014; Nicolas et al., 2018; Zhang et al., 2022). However, it is unclear whether the mechanical stress response is associated with the pathogenesis of neurodegeneration. Therefore, it is crucial to evaluate the cytoskeletal organization, FA formation and the mechanical stress response induced by arginine-rich poly-dipeptides.

In this study, we evaluated the effects of PR poly-dipeptides, the most toxic among five different poly-dipeptides (Kwon et al., 2014), on the cytoskeletal organization, cell stiffness, and FA formation. PR poly-dipeptides induced a high-density network of IFs and increased cell stiffness in conjunction with the abnormal remodeling of actin filaments and FA. PR poly-dipeptides prevented cell detachment. Furthermore, they induced the expression of mechanical stress response factors, leading to a maladaptive mechanical stress response. These results suggest that PR poly-dipeptides alter the stability of IFs, resulting in changes to mechanical stress responses.

## Materials and Methods

### Peptide Synthesis

A synthetic peptide consisting of twenty repeats of the PR poly-dipeptide (PR_20_) with an HA tag at the carboxyl terminus was synthesized (SCRUM Inc., Tokyo, JAPAN).

### Cell Culture

Human osteosarcoma cells (U2OS) and fibroblast (BJ-hTERT) cells were cultured in Dulbecco’s modified Eagle medium (DMEM) high glucose with 10% fetal bovine serum (FBS, Hyclone) and 1% penicillin-streptomycin at 37°C in 5% CO_2_. The cells were used for experiments after a one-hour treatment at 37°C with a synthetic peptide consisting of PR_20_ (final concentration 10 µM). Rat vascular smooth muscle cells (SMCs; Lonza, R-ASM-580) were grown in DMEM with 20% FBS and 1x Antibiotic-Antimyotic (Thermo Fisher Scientific).

### Immunofluorescence

The U2OS cells, fibroblast BJ-hTERT cells or rat vascular SMCs were fixed in 4% paraformaldehyde in phosphate-buffered saline (PBS) at room temperature for 15 min and permeabilized with 0.1% Triton X-100 in PBS for 10 min. The fixed cells were incubated with a blocking solution (5% bovine serum albumin in PBS with 0.1% Tween20) at room temperature for 1 h. The cells were incubated with primary antibodies, vimentin (Santa Cruz Biotechnology, sc6260, 1:100), cytokeratin 14 (Abcam, ab7800, 1:250), vinculin (Abcam, ab129002, 1:500), phospho-Paxillin (Cell Signaling, 2541, 1:50), phospho-ERM (Cell Signaling, 3726, 1:200), or phospho-Cofilin (Cell Signaling, 3313, 1:100) in the blocking solution at 4°C overnight. Secondary antibodies (Thermo Fisher Scientific, A-21422, A-21429, 1:2000) were incubated at room temperature for 1 h in the blocking solution. Alexa Fluor488-phalloidin (Thermo Fisher Scientific, A12379, 1:1000) and Alexa Fluor555-β-tubulin (Abcam, ab206627, 1:1000) were also incubated at room temperature for an hour in the blocking solution. Images were captured using the confocal microscope FV3000 (Olympus, Tokyo) or LSM 710 (ZEISS). Captured images were analyzed by ImageJ (version 1.53d, National Institutes of Health (NIH), United States).

### Imaging Analysis

Analysis of IF organization was performed by quantifying the junctions, branches, and alignments. The images were converted to 8-bit and binarized using fixed contrast and brightness thresholds. For junction and branch measurements, 100 μm^2^ regions of interest (ROI) in the cytoplasm, excluding the nucleus, were selected. At least one ROI was selected per cell. The number of junctions and branches was measured in the skeletonized images using the skeleton plugin (https://imagej.net/AnalyzeSkeleton, last accessed 1 March 2022). The bundle alignments of IFs were measured by the dispersion of the IF angles in whole cells (Laly et al., 2021), using the OrientationJ plugin (http://bigwww.epfl.ch/demo/orientation/, last accessed 1 March 2022). Fluorescence intensity of IFs in perinuclear regions was measured within 1 μm from the nucleus. Fluorescence intensity of actin filaments was evaluated by the Quimp plugin, which outlines a cell using their original algorithm (Baniukiewicz et al., 2018). Based on the outline, the plugin measures the fluorescence intensity of the whole cell and around the cell cortex. As the fluorescence intensity around the cell cortex is affected by the size of the cell, the ratio of fluorescence intensity around the cell cortex to the fluorescence intensity of the whole cell was calculated. To measure the fluorescence intensity of pERM, a region of cells was defined in the merged image. The average fluorescence intensity of this region was measured on the pERM image. The average fluorescence intensity was divided by the number of cells in the ROI to obtain the fluorescence intensity of pERM per cell.

Analysis of FA was performed by quantifying the size and the number of FA. The images were converted to 8-bit and binarized using fixed contrast and brightness thresholds. The average size and the number of FAs per cell were quantified from binary images using the “Analyze Particle” function in ImageJ (https://imagej.net/imaging/particle-analysis, last accessed 1 March 2022) with the following settings: size (pixel^2), 0.5-infinity; circularity, 0.00–1.00. The polymerized β-tubulin was measured using the Tubeness plugin (https://imagej.net/plugins/tubeness, last accessed 1 March 2022), which identified tube-like structures. The ratio of the polymerized β-tubulin to cell area was calculated.

### Atomic Force Microscopy

Atomic force microscopy (AFM) measurements were performed using a NanoWizard IV AFM (JPK Instruments-AG, Germany) mounted on top of an inverted optical microscope (IX73, Olympus, Japan) equipped with a digital CMOS camera (Zyla, Andor) as described in a previous study (Nagayama et al., 2019). Prior to AFM imaging of the surface topography and mechanical properties of U2OS cells in PR_20_-treated cells, the cells were adapted to a CO_2_-independent medium (Invitrogen) for 30 min at room temperature (25 °C). AFM quantitative imaging (QI) mode was used to obtain a force–displacement curve at a resolution of 128 × 128 pixels (100 µm × 100 µm of measured area) by a precisely controlled high-speed indentation test using rectangular-shaped silicon nitride cantilevers with a cone probe (BioLever-mini, BL-AC40TS-C2, Olympus, Japan). The test was performed at a spring constant of 0.08–0.10 N/m and a nominal tip radius of 10 nm. The QI mode measurements were performed within an hour after the transfer of the specimen to the AFM. These high-speed indentations were performed until a preset force of 1 nN was reached. This typically corresponded to cell indentation depths of 300–400 nm. Cell elasticity was calculated from the obtained force–displacement curves by applying the Hertzian model (Hertz, 1881), which approximates the sample to be isotropic and linearly elastic. Young’s (elastic) modulus is extracted by fitting all force–displacement curves with the following Hertzian model approximation:

$$F=\frac{2E\cdot \tan\alpha }{\pi \left(1-v^{2}\right)}\delta^{2}$$

where F is the applied force, E is the elastic modulus, ν is the Poisson’s ratio (0.5 for a non-compressible biological sample), α is the opening angle of the cone of the cantilever tip, and δ is the indentation depth of the sample recorded in the force–displacement curves. Using the results of the Hertzian model approximation, we identified the Z contact points (specimen surface) and the elastic modulus of the specimens at each pixel and produced a surface topography map and elastic modulus map of the specimens.

### Intracellular Calcium Imaging

The intracellular calcium concentration was analyzed using the Calbryte590™ assay kit (AAT Bioquest, United States). The U2OS cells were seeded in 96-well glass bottom microplates and cultured for 24 h. The cells were incubated for an hour at 37°C with Calbryte590™. After removal of the Calbryte590™ solution, the cells were administrated to 10 µM of PR_20_ for an hour and analyzed by a confocal microscopy and SpectraMax microplate reader (Molecular Devices, United States).

### Cell Detachment Experiment

The U2OS cells were transfected with LifeAct-GFP plasmids (60101, ibidi GmbH) using FuGENE-HD transfection reagent (E2311, Promega). For the cell detachment experiment, transfected cells were seeded in glass bottom plates and cultured for 24 h. The cells were administered to 10 µM of PR_20_ for an hour, 50 μM of (-)-Blebbistatin (B0560-1MG, Sigma-Aldrich) for an hour (Korobova et al., 2014), 10 µM of Nocodazole (M1404-10MG, Sigma-Aldrich) for 30 min (Baum et al., 2014), 2 µM of Withaferin-A (ab120644, Abcam) for 3 hours (Grin et al., 2012), and observed after 30 min incubation with 5 µM of EDTA. EDTA reduces integrin-mediated adhesion, which is a Ca^2+^-dependent FA molecule by chelating Ca^2+^ and Mg^2+^, causing cell detachment (Mould et al., 1995). Equivalent amounts of DMSO (D8418, Sigma–Aldrich) were added as vehicle control, as Blebbistatin, Nocodazole and Withaferin-A were diluted with DMSO. Blebbistatin, Nocodazole and Withaferin-A are used as cytoskeleton inhibitors. Blebbistatin is an inhibitor of myosinⅡ and inhibits the formation of actomyosin and suppresses stress fiber formation (Kovács et al., 2004). Nocodazole binds to the growing end of β-tubulin and prevents microtubule polymerization (Chang et al., 2008). Withaferin-A binds to the vimentin rod domain and disrupts both Vim-IF and Keratin-IF network (Bargagna-Mohan et al., 2007; Grin et al., 2012). Images were captured using a confocal microscope FV3000 (Olympus, Tokyo). Captured images were analyzed by ImageJ (version 1.53d, NIH, United States). As detached cells with a round shape showed high circularity (Fuhrmann et al., 2014), degrees of cell detachment were analyzed by measuring the circularity of cells.

### Cyclic Stretch Experiment

Cyclic stretch was performed using a uniaxial cell stretch system (Central Workshop Tsukuba University) as described in a previous study (Yamashiro et al., 2020). We employed rat vascular smooth muscle cells (SMCs) or U2OS cells. The cells were plated on silicon elastomer bottomed culture plates (SC4Ha, Menicon Life Science) coated with a cell attachment factor containing gelatin (Thermo Fisher Scientific, S006100) and subjected to cyclic stretch with a frequency of 1.0 Hz (60 cycles/min) and 20% strain for 6 h.

### Western Blotting

Total proteins are isolated from rat vascular SMCs treated with 10 µM of PR_20_ using RIPA lysis buffer. After sodium dodecyl sulphate–polyacrylamide gel electrophoresis (SDS-PAGE), proteins were transferred to polyvinylidene difluoride membranes (PVDF). Membranes were blocked with and incubated overnight at 4°C with the following primary antibodies: Thbs1 (NeoMarkers, MS-421), Egr1 (Cell Signaling, 4154), phospho-FAK(Y397) (Cell Signaling, 3283), FAK (Cell Signaling, 3285), phospho-ERK (Cell Signaling, 4376), ERK (Cell Signaling, 9102), phospho-ERM (Cell Signaling, 3141), ERM (Cell Signaling, 3142), GAPDH (Cell Signaling, 2118). Then, membranes were incubated with second antibodies anti-mouse or anti-rabbit HRP-conjugated antibody (1:1000, Bio-Rad). Blots were visualized using a chemiluminescence kit (Santa Cruz Biotechnology) or SuperSignal West Femto Maximum Sensitivity Substrate (Thermo Fisher Scientific).

### Statistical Analysis

All experiments are presented as means ± SD (except for Figure 6B). Statistical analysis was performed using Prism 8 (GraphPad Software, California, United States). A Shapiro-Wilk test was used to test for normality. The Mann–Whitney U test, a nonparametric test, or unpaired *t* test was conducted. *p* < 0.05 denotes statistical significance.

## Results

### PR Poly-Dipeptides Increase the Junctions and Branches of the Intermediate Filament Network

**FIGURE 1:**
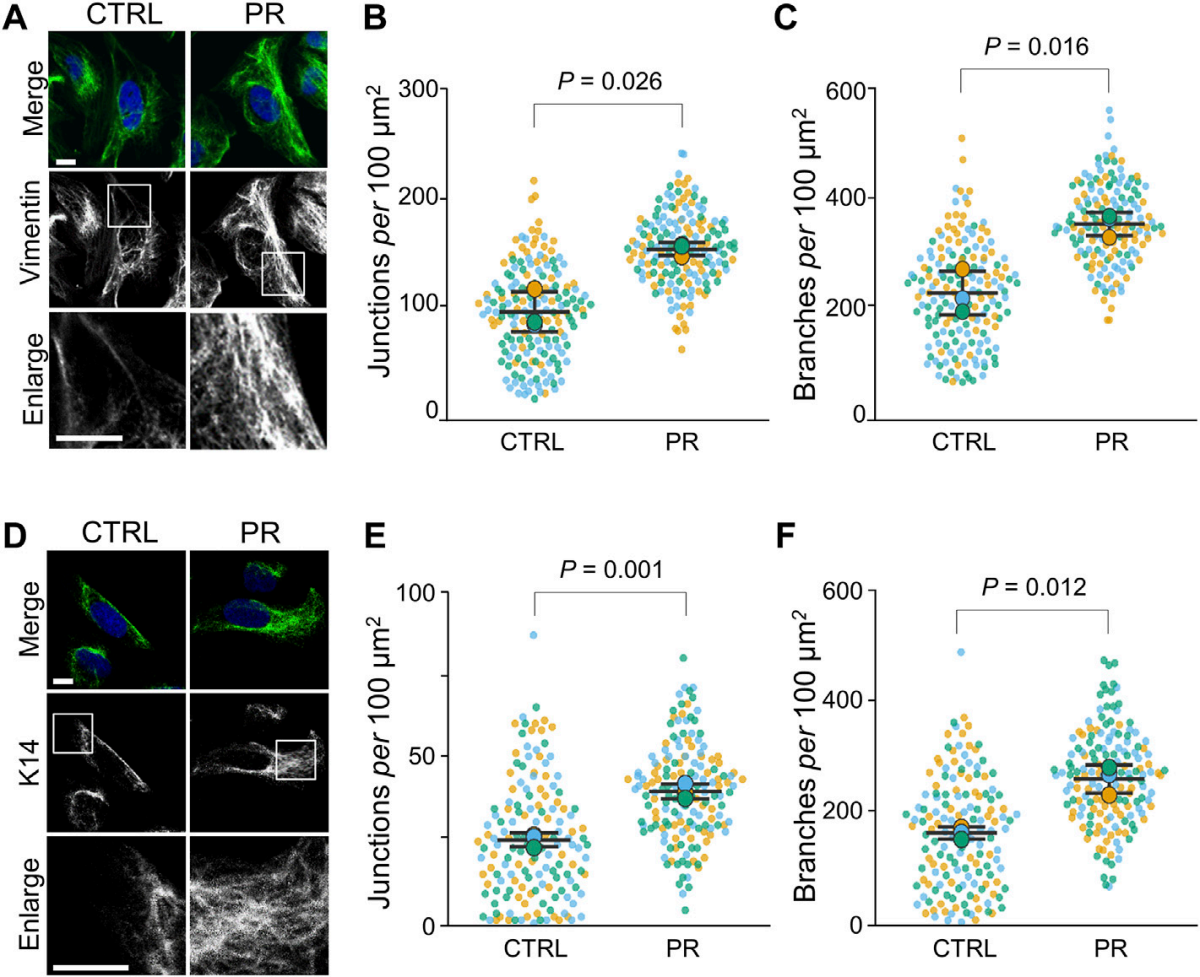
PR poly-dipeptides (PR_20_) change the organization of intermediate filaments (IF) of U2OS cells. **(A)** Fluorescent images of Vim-IFs in untreated control (CTRL) and PR_20_-treated (PR) cells. Vim-IFs (green) and DAPI (blue) are shown. Scale bars are 10 µm. **(B,C)** Quantification of Vim-IF junctions (in B) and branches (in C) per 100 μm^2^ in the cytoplasm in CTRL and PR cells. Bars are means ± SD. In each experiment, 50 to 60 cells were evaluated (*n* = 3). P value shows Welch’s *t*-test. **(D)** Fluorescent images of cytokeratin14 (K14) in CTRL and PR cells. K14 (green) and DAPI (blue) are shown. Scale bars are 10 µm. **(E,F)** Quantification of Keratin-IF junctions (in E) and branches (in F) per 100 μm^2^ in the cytoplasm in CTRL and PR cells. Bars are means ± SD. In each experiment, 50 to 58 cells were evaluated (*n* = 3). *p* value shows Welch’s *t*-test. In quantification graphs, small dots are individual datapoints, and large dots indicate the averages of each experiment in orange (first; *n* = 1), blue (second; *n* = 2) and green (third; *n* = 3), respectively.

To investigate the effect of PR poly-dipeptides on cytoskeleton, we first examined the changes of Vimentin (Vim) and Keratin intermediate filament organization in U2OS cells. Vim-IFs were predominantly localized to the perinuclear region in the control cells (CTRL; vehicle control) (Figure 1A). After 1 h of exposure to 10 µM of PR_20_, the fluorescence intensity of Vim-IFs in the perinuclear region decreased (*p* = 0.028; 19.8 ± 2.8 a.u. in CTRL, 12.6 ± 1.5 a.u. in PR_20_-treated; Supplementary Figure S1A) and Vim-IFs formed a mesh network in the cytoplasm (Figure 1A). The IF organization was analyzed by the alignments and the number of branches and junctions in skeletonized images (Supplementary Figure S1B). Vim-IF alignments were comparable between CTRL and PR_20_-treated cells (*p* = 0.678; 21.0 ± 2.2 in CTRL, 21.9 ± 2.5 in PR_20_-treated; Supplementary Figure S1C). There were more junctions of Vim-IFs in PR_20_-treated cells than in CTRL (*p* = 0.026; 97.3 ± 18.1 in CTRL, 154.5 ± 5.9 in PR_20_-treated; Figure 1B). There were also more branches of Vim-IFs in PR_20_-treated cells than in CTRL (*p* = 0.016; 232.9 ± 39.5 in CTRL, 358.2 ± 21.0 in PR_20_-treated; Figure 1C).

Next, Keratin-IFs were analyzed using cells stained with anti-cytokeratin14 (K14) antibody. Keratin-IFs formed a mesh network in the cytoplasm of PR_20_-treated cells (Figure 1D). The fluorescence intensity of Keratin-IFs in the perinuclear region was comparable between CTRL and PR_20_-treated cells (*p* = 0.428; 12.7 ± 1.3 in CTRL, 13.9 ± 2.2 in PR_20_-treated; Supplementary Figure S1D). Keratin-IF alignments were also comparable between CTRL and PR_20_-treated cells (*p* = 0.919; 44.7 ± 3.6 in CTRL, 45.1 ± 5.2 in PR_20_-treated; Supplementary Figure S1E). There were more junctions (*p* = 0.001; 25.5 ± 2.1 in CTRL, 40.3 ± 2.3 in PR_20_-treated) and branches (*p* = 0.012; 165.9 ± 11.0 in CTRL, 264.3 ± 26.0 in PR_20_-treated) of Keratin-IFs in PR_20_-treated cells than in CTRL (Figures 1E,F). Together, these results indicate that PR poly-dipeptides induce a high-density network of IFs in the cytoplasm.

### PR Poly-Dipeptides Increase Cell Stiffness

**FIGURE 2:**
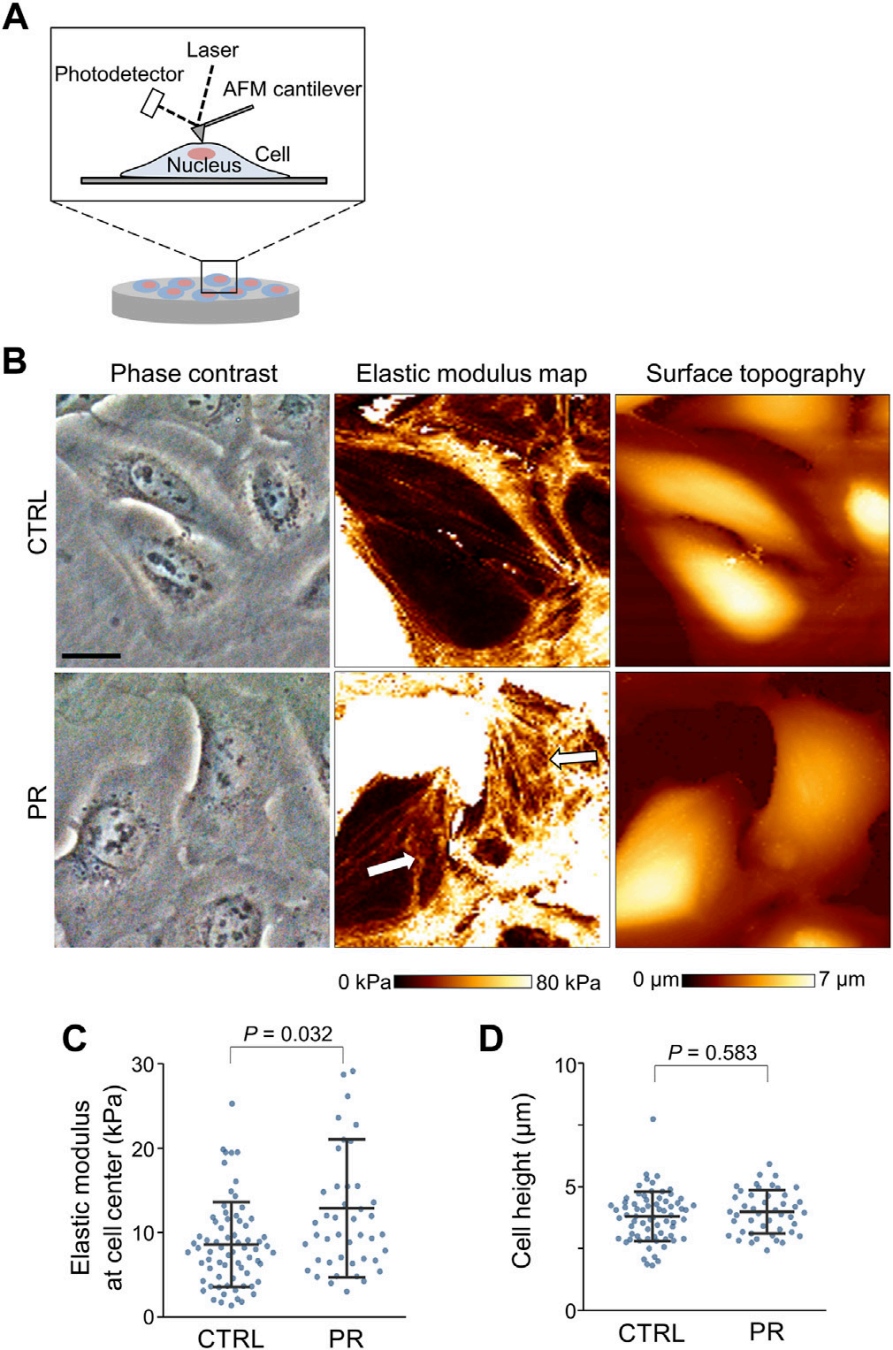
Atomic force microscopy (AFM) images of the surface of U2OS cells. **(A)** Schematic view of AFM measurement of cell surface, including cell stiffness and cell height. **(B)** Representative images of phase contrast images (left), elastic modulus maps (middle), and surface topographic images (right) of U2OS cells measured by AFM. Arrows in the middle image show thick- and short-stress fibers in PR_20_-treated (PR) cells. Scale bars are 20 µm. **(C,D)** Quantification graph shows elastic modulus at cell central region (in C) and cell height (in D) in CTRL and PR cells. Bars are means ± SD. 45 to 69. Cells were evaluated per condition. P value shows Mann–Whitney U test.

To evaluate the effects of PR poly-dipeptides on cellular mechanics, we analyzed the cell stiffness and cell height by atomic force microscopy (AFM) in U2OS cells (Figure 2A). The number of filamentous actin (F-actin) stress fibers across the whole cell was reduced, and F-actin stress fibers around the nucleus were thick and short in PR_20_-treated cells (Figure 2B, middle, arrows). We measured the average elastic modulus at the cell central region. Cell elasticity at the cell central region increased in PR_20_-treated cells than in CTRL (*p* = 0.032; 8.6 ± 5.1 kPa in CTRL, 12.9 ± 8.2 kPa in PR_20_-treated; Figure 2C). There were no significant differences in cell height between CTRL and PR_20_-treated cells (*p* = 0.583; 3.9 ± 1.0 µm in CTRL, 4.0 ± 0.9 µm in PR_20_-treated; Figure 2D). These results suggest that PR poly-dipeptides increase cell stiffness by thick and short F-actin stress fibers.

### PR Poly-Dipeptides Change the Distribution of F-Actin

**FIGURE 3:**
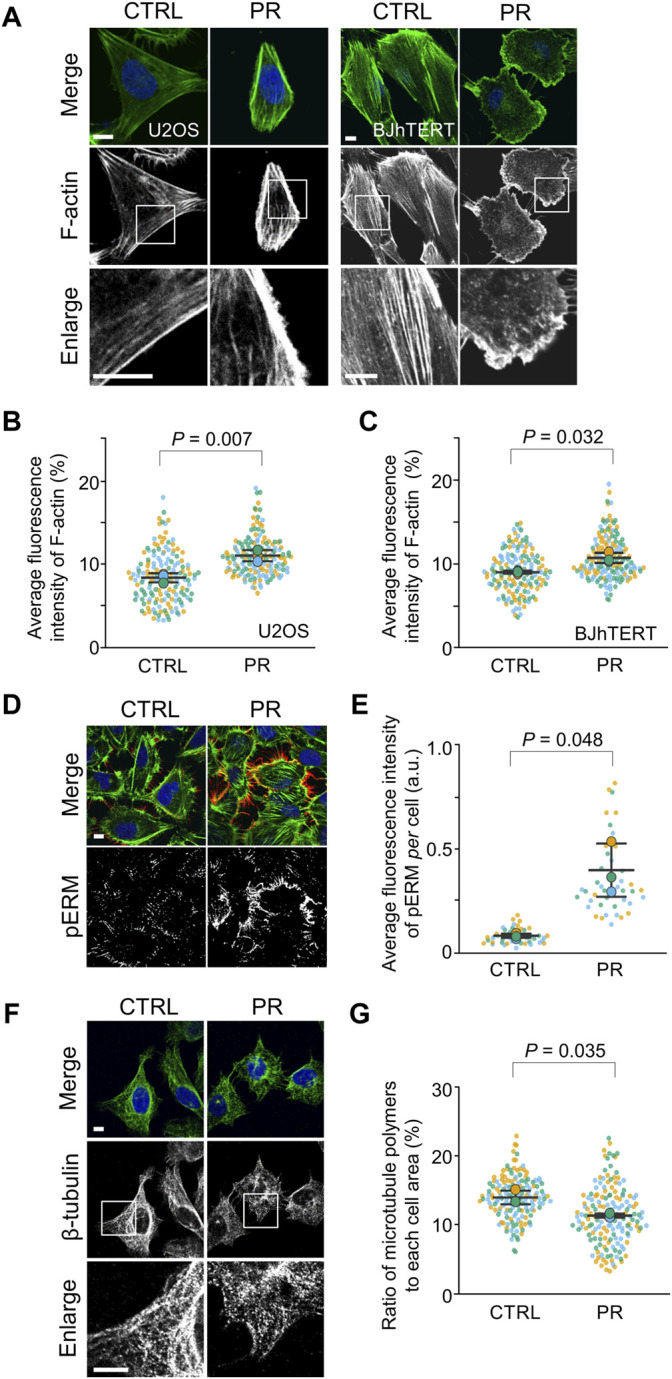
PR_20_ changes the organization of F-actin and β-tubulin and promotes the phosphorylation of ERM (pERM). **(A)** Fluorescent images of phalloidin in U2OS and BJhTERT cells after treatment with 10 µM of PR_20_ or CTRL. phalloidin (green) and DAPI (blue) are shown. Scale bars are 10 µm. **(B,C)** Quantification of average fluorescence intensity of F-actin in U2OS cells (in B) and BJhTERT cells (in C). Bars are means ± SD. In each experiment, 50 to 55 cells were evaluated (*n* = 3). *p* value shows Welch’s *t*-test. **(D)** Fluorescent images of pERM (red), phalloidin (green) and DAPI (blue) after treatment with or without 10 µM of PR_20_ in U2OS cells. A bar is 10 µm. **(E)** Average fluorescence intensity of pERM per cell after treatment with 10 µM of PR_20_ or CTRL. Bars are means ± SD. 15 regions were evaluated (*n* = 3). *p* value shows Welch’s *t*-test. **(F)** Fluorescent images of β-tubulin in U2OS cells after treatment with 10 µM of PR_20_ or CTRL. Bars are 10 µm. **(G)** The ratio of microtubule polymers in CTRL and PR_20_-treated (PR) cells. Bars are means ± SD. In each experiment, 50 to 57 cells were evaluated (*n* = 3). *p* value shows Welch’s *t*-test. In quantification graphs, small dots are individual datapoints, and large dots indicate the averages of each experiment in orange (first; *n* = 1), blue (second; *n* = 2) and green (third; *n* = 3), respectively.

To investigate the effects of PR poly-dipeptides on other cytoskeletal elements, we analyzed the distribution of F-actin by fluorescence images (Supplementary Figure S2A). In CTRL, F-actin extended across the whole cell and equally distributed in the cytoplasm, whereas after PR_20_-treatment, F-actin was not distributed equally in the cytoplasm but mainly localized around the cell cortex in U2OS cells and BJhTERT cells (Figure 3A). PR_20_-treated cells showed a higher fluorescence intensity of F-actin around the cell cortex than CTRL in both cell lines (U2OS; *p* = 0.007; 8.4 ± 0.6%, in CTRL, 11.0 ± 0.7% in PR_20_-treated cells. BJhTERT; *p* = 0.032; 9.1 ± 0.2%, in CTRL, 10.8 ± 0.6% in PR_20_-treated cells; Figures 3B,C). Similar to U2OS cells and BJhTERT cells (Figure 3A), PR_20_ changed F-actin organization in rat vascular smooth muscle cells (SMCs) (Supplementary Figure S2B). PR_20_ also caused nuclear deformation and the unclear boundary between the nucleus and the endoplasmic reticulum (ER) in SMCs (Supplementary Figure S2C). These results suggest that PR poly-dipeptides change the distribution of F-actin.

To evaluate whether the redistribution of F-actin by PR poly-dipeptides result from the F-actin reorganization, we analyzed Ezrin/Radixin/Moesin (ERM) proteins and Cofilin. As cortical F-actin is formed by ERM proteins (Bretscher et al., 2002), we analyzed cortical F-actin by ERM phosphorylation in U2OS cells (Supplementary Figure S2D). Phospho-ERM (pERM) localized to protrusive structures, such as filopodia, both in CTRL and PR_20_-treated cells (Figure 3D). The fluorescence intensity of pERM per cell was increased in PR_20_-treated cells (*p* = 0.048; 0.1 ± 0.0 a.u. in CTRL, 0.4 ± 0.1 a.u. in PR_20_-treated; Figure 3E). As F-actin is depolymerized by phosphorylated Cofilin (pCofilin) (Sumi et al., 1999), we evaluated pCofilin. The level of pCofilin was comparable between CTRL and PR_20_-treated cells in U2OS cells and SMCs (Supplementary Figures S2E,F). These results suggest that PR poly-dipeptides enhance the reorganization of cortical F-actin through pERM.

To evaluate the effects of PR poly-dipeptides on microtubules, we analyzed the ratio of polymerized β-tubulin. In CTRL, β-tubulin showed a filamentous network in the cytoplasm, but it appeared as dots with a diffused network in PR_20_-treated cells (Figure 3F). Compared to CTRL, PR_20_-treatment reduced the ratio of polymerized β-tubulin (*p* = 0.035; 13.9 ± 1.0% in CTRL, 11.3 ± 0.3% in PR_20_-treated cells; Figure 3G). These results suggest that PR poly-dipeptides disrupt the stability of the microtubule network.

### PR Poly-Dipeptides Increase FA Size and Intracellular Calcium Concentration

**FIGURE 4:**
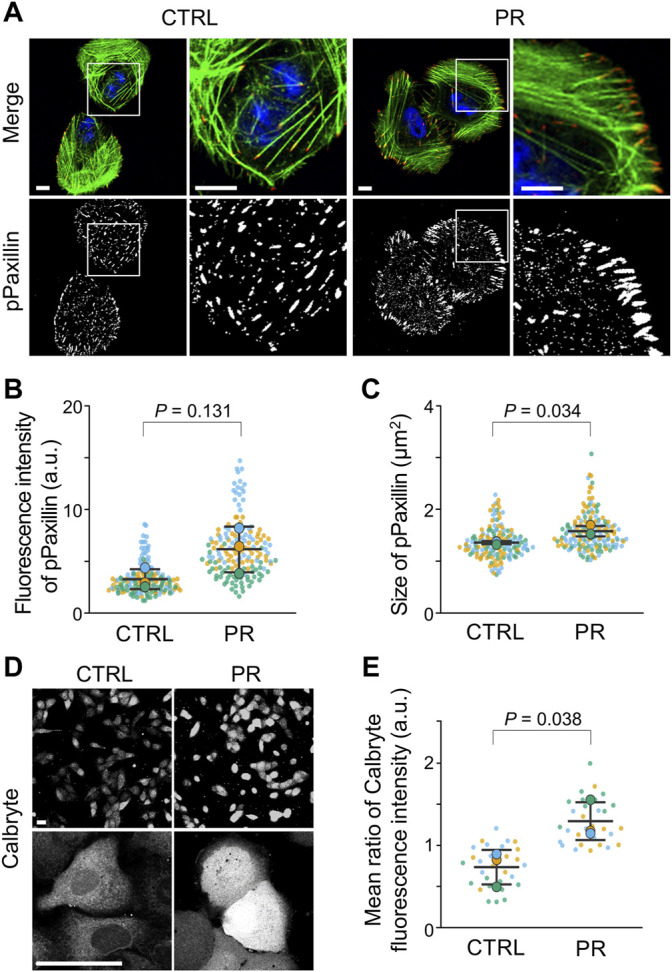
PR_20_ increases the size of FA and intracellular calcium concentration. **(A)** Fluorescent images of pPaxillin (red), phalloidin (green) and DAPI (blue) are shown. Scale bars are 10 µm. **(B,C)** Average fluorescence intensity of pPaxillin (in B) and size of pPaxillin (in C). Bars are means ± SD. In each experiment, 41 to 57 cells were evaluated (*n* = 3). *p* value shows Welch’s *t*-test. **(D,E)** Fluorescence images of Calbryte590™ in CTRL and PR_20_-treated (PR) cells. Scale bars are 50 µm. Quantification shows in E. Bars are means ± SD. In each experiment, 10 cells were evaluated (*n* = 3). *p* value shows Welch’s *t*-test. In quantification graphs, small dots are individual datapoints, and large dots indicate the averages of each experiment by orange (first; *n* = 1), blue (second; *n* = 2), and green (third; *n* = 3), respectively.

To investigate the effects of PR poly-dipeptides on FA formation, we analyzed the localization, the number and size of Vinculin and pPaxillin in U2OS cells (Supplementary Figure S3A). In CTRL, we found Vinculin and pPaxillin to be equally distributed in the cytoplasm (Supplementary Figure S3B; Figure 4A). On the other hand, in PR_20_-treated cells, Vinculin and pPaxillin were reduced in the cell central region and mainly observed in the cell periphery (Supplementary Figure S3B; Figure 4A). Although the number of Vinculin decreased in PR_20_-treated cells (*p* = 0.003; 68.9 ± 3.6 in CTRL, 36.6 ± 6.2 in PR_20_-treated; Supplementary Figure S3C), the size of Vinculin was comparable between CTRL and PR_20_-treated cells (*p* = 0.506; 1.6 ± 0.1 µm^2^ in CTRL, 1.7 ± 0.3 µm^2^ in PR_20_-treated; Supplementary Figure S3D). There was no significant difference in the fluorescence intensity of pPaxillin between CTRL and PR_20_-treated cells (*p* = 0.131; 3.3 ± 1.0 a.u. in CTRL, 6.2 ± 2.2 a.u. in PR_20_-treated; Figure 4B). However, the size of pPaxillin was larger in PR_20_-treated cells than in CTRL (*p* = 0.034; 1.4 ± 0.0 µm^2^ in CTRL, 1.6 ± 0.1 µm^2^ in PR_20_-treated; Figure 4C). These results suggest that PR poly-dipeptides induce the maturation of FA.

Since FA size is regulated by intracellular calcium (Huang et al., 2018), we measured the intracellular calcium concentration using calcium indicator calbryte590™ (Supplementary Figure S3E). The fluorescent images of PR_20_-treated cells showed a stronger fluorescence signal compared to CTRL cells (Figure 4D). The calbryte590™ fluorescence intensity using a microplate reader was higher in PR_20_-treated cells than in CTRL (*p* = 0.038; 0.7 ± 0.2 in CTRL, 1.3 ± 0.2 a.u. in PR_20_-treated; Figure 4E). These results suggest that PR poly-dipeptides increase intracellular calcium concentration.

### PR Poly-Dipeptides Prevent Cell Detachment

**FIGURE 5:**
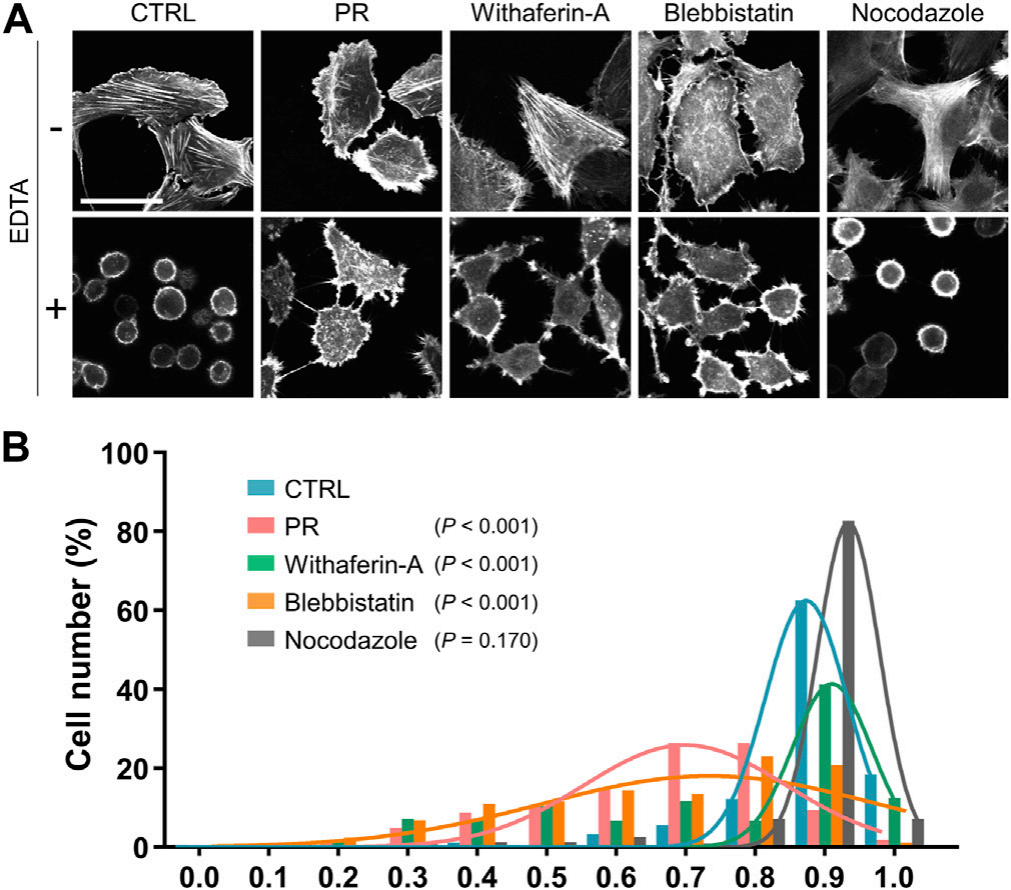
PR_20_ inhibits cell detachment by EDTA. **(A)** Representative LifeAct-GFP images of U2OS cells in CTRL and PR_20_-treated (PR) and the cytoskeletal inhibitors, Withaferin-A, Blebbistatin and Nocodazole treatment, following detachment by 5 µM EDTA. A bar is 50 µm. **(B)** A histogram of the cell circularity of U2OS cells after detachment by 5 µM EDTA in CTRL (*n* = 179, blue), PR_20_ (*n* = 158, red), Withaferin-A (*n* = 243, green), Blebbistatin (*n* = 316, orange), and Nocodazole *(n* = 242, gray). A circularity of 1.0 indicates a complete circle. As it approaches 0.0, it indicates an elongated polygon. *p* value shows Welch’s *t*-test.

To investigate the impact of cytoskeletal organization on adhesive strength, U2OS cells were transfected with Lifeact-GFP, which enables live imaging of F-actin, and challenged by cell detachment using EDTA after PR poly-dipeptides and cytoskeleton inhibitors, Withaferin-A, Blebbistatin and Nocodazole. Before EDTA treatment, F-actin stress fibers extended across the whole cell in CTRL. PR_20_-treated cells enhanced cortical F-actin compared to CTRL (Figure 5A), while Withaferin-A-treated cells were comparable to CTRL (Figure 5A). Blebbistatin-treated cells showed fewer F-actin stress fibers than CTRL (Figure 5A). Nocodazole-treated cells showed more F-actin stress fibers than CTRL (Figure 5A). After EDTA-treatment, CTRL and Nocodazole-treated cells showed a rounded shape and F-actin was localized in the cell cortex, whereas PR_20_, Withaferin-A and Blebbistatin-treated cells maintained a spread shape and formed protrusions (Figure 5A).

As the detached cells showed a rounded shape and high circularity (Fuhrmann et al., 2014), degrees of cell detachment were analyzed by measuring the circularity of cells. The circularity of PR_20_-treated cells was lower than CTRL (*p* < 0.001; 0.9 ± 0.1 in CTRL, 0.7 ± 0.2 in PR_20_; Figure 5B; Supplementary Figure S4). Withaferin-A-treated cells (*p* < 0.001; 0.8 ± 0.0) and Blebbistatin-treated cells (*p* < 0.001; 0.7 ± 0.2) also decreased circularity after EDTA-treatment (Figure 5B; Supplementary Figure S4). Nocodazole-treated cells showed no significant difference in the circularity compared to CTRL after EDTA-treatment (*p* = 0.170; 0.9 ± 0.0 in Nocodazole; Figure 5B; Supplementary Figure S4). These results indicate that PR poly-dipeptides prevent cell detachment via the reorganization of IFs and F-actin.

### PR Poly-Dipeptides Attenuate the Cyclic Stretch-Induced Reorientation of F-Actin Stress Fibers

**FIGURE 6:**
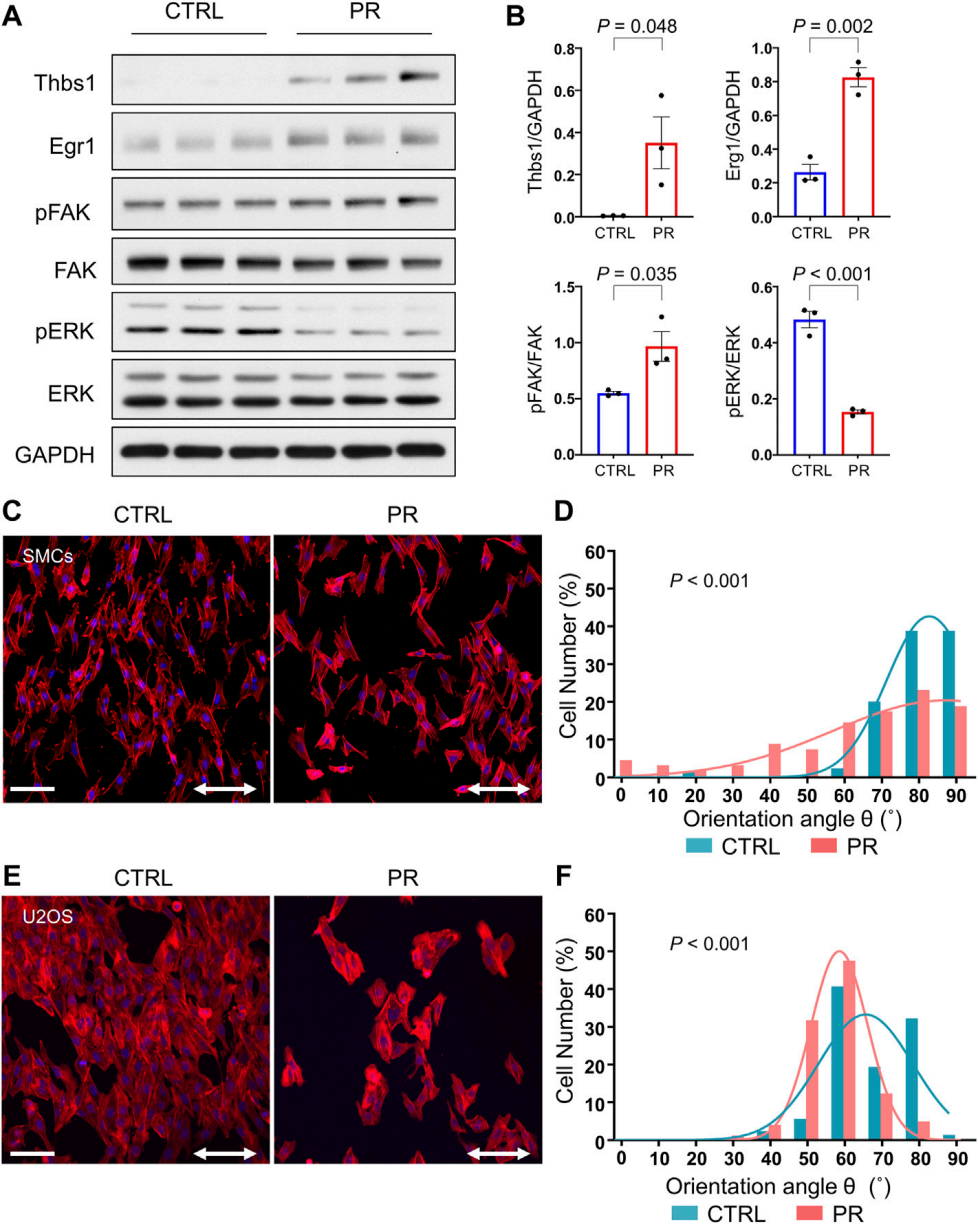
PR_20_ prohibits the cyclic stretch-induced reorientation of F-actin stress fibers. **(A)** Western blotting shows Thbs1, Egr1, pFAK, FAK, pERK and ERK levels in CTRL and PR_20_-treated (PR) cells (*n* = 3). GAPDH was used as a loading control. **(B)** Quantification graphs are shown. Bars are means ± SEM. *p* value shows unpaired *t*-test. **(C,E)** Rat vascular SMCs (in C) and U2OS cells (in E) with or without 10 µM of PR_20_ were subjected to cyclic stretch (20% strain, 1.0 Hz (60 cycles/min) for 6 hours. Phalloidin (red) and DAPI (blue) are shown. Scale bars are 100 µm. The two-way arrows indicate stretch direction. **(D,F)** Histograms of the percentage of the orientation angle (θ) for each cell in rat vascular SMCs (in D) and U2OS cells (in F). The orientation of each cell was analyzed by measuring the orientation angle (θ) of the long axis of the ellipse relative to the stretch axis in CTRL (blue) and PR cells (red). 70 to 104 cells were evaluated in each condition. *p* value shows Mann–Whitney U test.

Thus far, we have observed that PR poly-dipeptides induced changes in cytoskeletal organization and FA formation, leading to the increase in cell stiffness and the prevention of cell detachment. Therefore, we assume that PR poly-dipeptides might alter the mechanical stress response. To test this hypothesis, we used rat SMCs and U2OS cells and performed uniaxial stretch experiment. Mechanical stretch responses were evaluated by the reorientation angle and the expression of mechanical stress response factor, such as early growth response 1 (Egr1), Thbs1, phosphorylation of extracellular signal-regulated kinase (ERK) and FAK as previously reported (Yamashiro et al., 2015; Yamashiro et al., 2018; Yamashiro et al., 2020). Interestingly, in rat SMCs, PR_20_-treatment activated FA and upregulated the expression of mechanical stress response factors prior to stretch-stimuli (Figures 6A,B). Although CTRL cells were aligned perpendicularly to stretch direction, PR_20_-treated cells failed to align correctly and decreased number of cells after stretching (Figure 6C). A histogram of the percentage of the orientation angle (θ) showed that PR_20_ suppressed the cyclic stretch-induced reorientation of F-actin stress fibers (*p* < 0.001, 75.3 ± 10.5° in CTRL, 59.4 ± 23.2° in PR_20_; Figure 6D). U2OS cells showed similar results as rat SMCs (*p* < 0.001, 73.1 ± 10.7° in CTRL, 65.8 ± 8.8° in PR_20_; Figures 6E,F). Consequently, these results suggest that PR poly-dipeptides promote the activation of FA and induce upregulation of mechanical stress response factors, leading to sensitive and maladaptive responses to mechanical stimuli.

## Discussion

Here we show that PR poly-dipeptides increased the junctions and branches of the IF network and cell stiffness. This is in conjunction with changes to the distribution of F-actin and the enhancement of FA size. We also demonstrated that cytoskeletal reorganization induced by PR poly-dipeptides prevented cell detachment and led to maladaptive responses to cyclic stretch.

**FIGURE 7:**
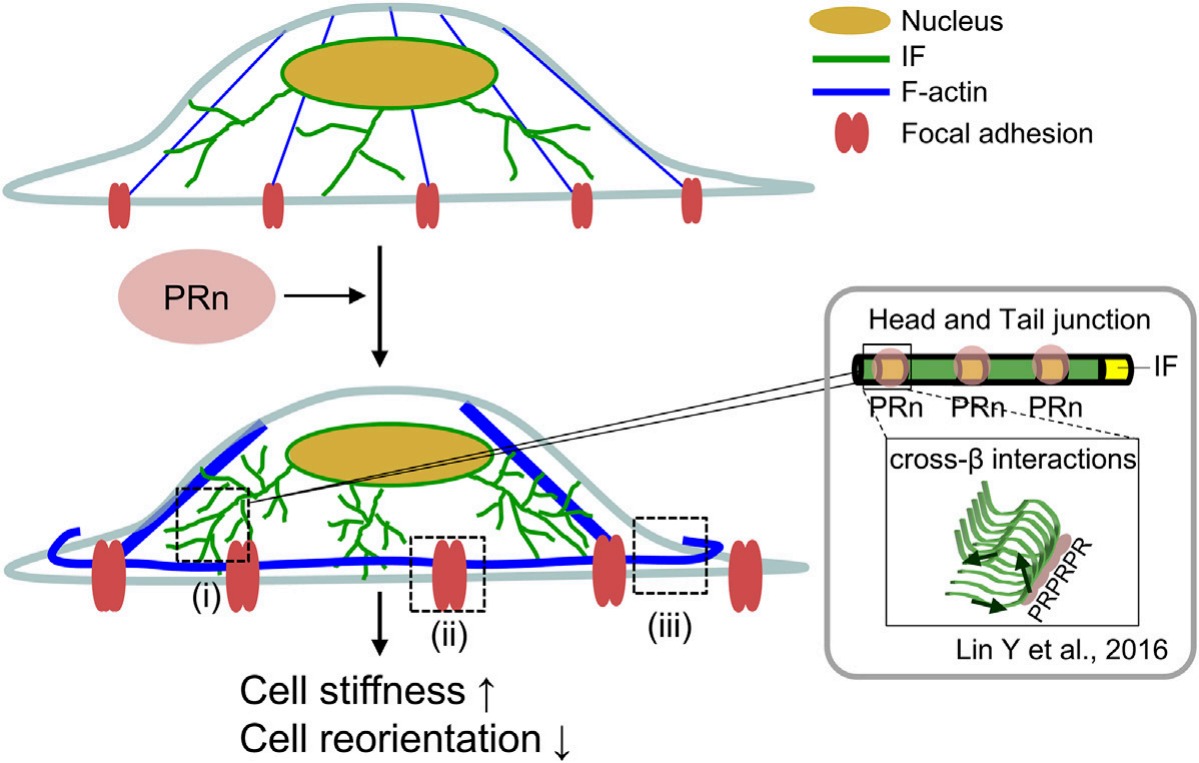
Graphical abstract. PR poly-dipeptides bind to IF head domain and induced the alterations of cellular mechanics. PR poly-dipeptides (pink) bind to the IF head domain (yellow) and dysregulate the IF dynamics. This results in an increase of IF branches and IF mesh network (green) (ⅰ). The IF network increases the size of FA (red) (ⅱ) and thickness of F-actin stress fibers (blue) (ⅲ), leading to cell stiffness. Further, the alterations of cytoskeleton and FA disrupt cell reorientation after cyclic stretch. IF: intermediate filament, PRn: PR poly-dipeptides.

PR poly-dipeptides bind proteins with low-complexity domains and nuclear import receptors (Lee et al., 2016; Lin et al., 2016; Hayes et al., 2020; Hutten et al., 2020; Nanaura et al., 2021). Head domains of IF proteins self-associate *via* the formation of labile but structurally specific cross-β interactions (Zhou et al., 2021), and PR poly-dipeptides target these polymeric forms of the IF head domains (Lin et al., 2016). In this study, we show that PR poly-dipeptides increased the junctions and branches of the IF network (Figure 1). We speculate that PR poly-dipeptides stabilize these dynamic cross-β interaction of the IF head domains, and lead to the malfunction and dysregulation of IF network formation (Figure 7). However, the detailed mechanisms of how PR poly-dipeptides interfere with cytoskeleton in cells remain unknown.

As the IF network contributes to cell stiffness (Laly et al., 2021), the high-density network of IFs induced by PR poly-dipeptides might increase cell stiffness. PR poly-dipeptides also reduced the fluorescence intensity of Vim-IFs in the perinuclear region but not Keratin-IFs (Figures 1A,D). These data might result from a difference in IF localization—Vim-IFs diffuse in the perinuclear region and the cytoplasm, whereas Keratin-IFs mainly distribute in the cytoplasm (Grin et al., 2012; Li et al., 2019).

PR poly-dipeptides induced the formation of cortical F-actin and the maturation of FA (Figures 3, 4). The formation of cortical F-actin and the maturation of FA are regulated by IF organization (Helfand et al., 2011). The IF organization by PR poly-dipeptides might alter the formation of cortical F-actin and the maturation of FA. Cortical F-actin is formed by ERM proteins (Bretscher et al., 2002). ERM proteins also induce FAK activation via signaling pathways independent of FA (Poullet et al., 2001). PR poly-dipeptides might induce the maturation of FA via ERM phosphorylation. Nonetheless, it is still unclear how PR poly-dipeptides affect the formation of cortical F-actin and the maturation of FA.

The dysregulation of intracellular calcium is reported as a cause of cell death in C9-ALS/FTD (Li et al., 2020; Choi et al., 2019; Dafinca et al., 2016; Dafinca et al., 2020; Baughman et al., 2011). PR poly-dipeptides increased the intracellular calcium concentration (Figures 4D,E). The increase in intracellular calcium concentration promotes cytoskeletal reorganization and FA maturation (Price et al., 2003; Hartzell et al., 2016; O'Brien et al., 1997). Cytoskeletal reorganization and FA maturation by PR poly-dipeptides might be related to the increase in intracellular calcium. Further investigation is required to understand how the increase in intracellular calcium by PR poly-dipeptides is related to cytoskeletal reorganization and FA maturation.

PR poly-dipeptides, Blebbistatin, and Withaferin-A prevented cell detachment (Figure 5). Cell detachment depends on the adhesive strength and cell stiffness (Sen and Kumar, 2009; Tee et al., 2011). While Blebbistatin decreases cell stiffness by myosin II inhibition (Sen and Kumar, 2009), Withaferin-A increases cell stiffness by inhibition of IF organization (Bargagna-Mohan et al., 2007). Taken together, the alteration of IF organization by PR poly-dipeptides might prevent cell detachment through increasing cell stiffness.

Further, we found that PR poly-dipeptides induced upregulation of Thbs1, a mechanical stress response factor (Figures 6A,B). Thbs1 is induced by cyclic stretch and is related to mechanical stress response (Yamashiro et al., 2020; Laly et al., 2021), suggesting that PR poly-dipeptides induce a maladaptive mechanical stress response in a Thbs1-dependent manner. For the adaptive mechanical stress response, the cells first reduce the adhesive strength of FA, and then they reorient to a perpendicular position and reorganize F-actin stress fibers and FA (Nagayama et al., 2012; Livne et al., 2014). The maturation of FA by PR poly-dipeptides increases adhesive strength and might consequently suppress cell reorientation after cyclic stretch. The mechanical stress response regulates a wide range of cellular functions involved in cell death (King et al., 2011). Hence, further research is required to investigate the effect of maladaptive mechanical stress responses induced by PR poly-dipeptides on intracellular signaling.

PR poly-dipeptides reduced the number of cells after cyclic stretch (Figures 6C,E). PR poly-dipeptides cause cell death in a time-dependent manner (Kwon et al., 2014), resulting in the reduction of the number of cells. In addition, by FA maturation, cells treated with PR poly-dipeptides are expected to be exposed to higher continuous mechanical stress during cyclic stretch, resulting in the reduction of number of cells.

This study revealed that PR poly-dipeptides change the IF organization, leading to an alteration of the mechanical stress response. These results suggest that the pathogenesis by arginine-rich poly-dipeptides might be partially explained by the changes in cellular mechanical properties and mechanical stress responses.

## Data Availability

The raw data supporting the conclusions of this article will be made available by the authors, without undue reservation.
