# Supplementary figures and images for: C9orf72-Derived Proline:Arginine Poly-Dipeptides Modulate Cytoskeleton and Mechanical Stress Response
Source: Front Cell Dev Biol. 2022 Mar 23;10:750829. doi: 10.3389/fcell.2022.750829 (PMC8983821; doi:10.3389/fcell.2022.750829)

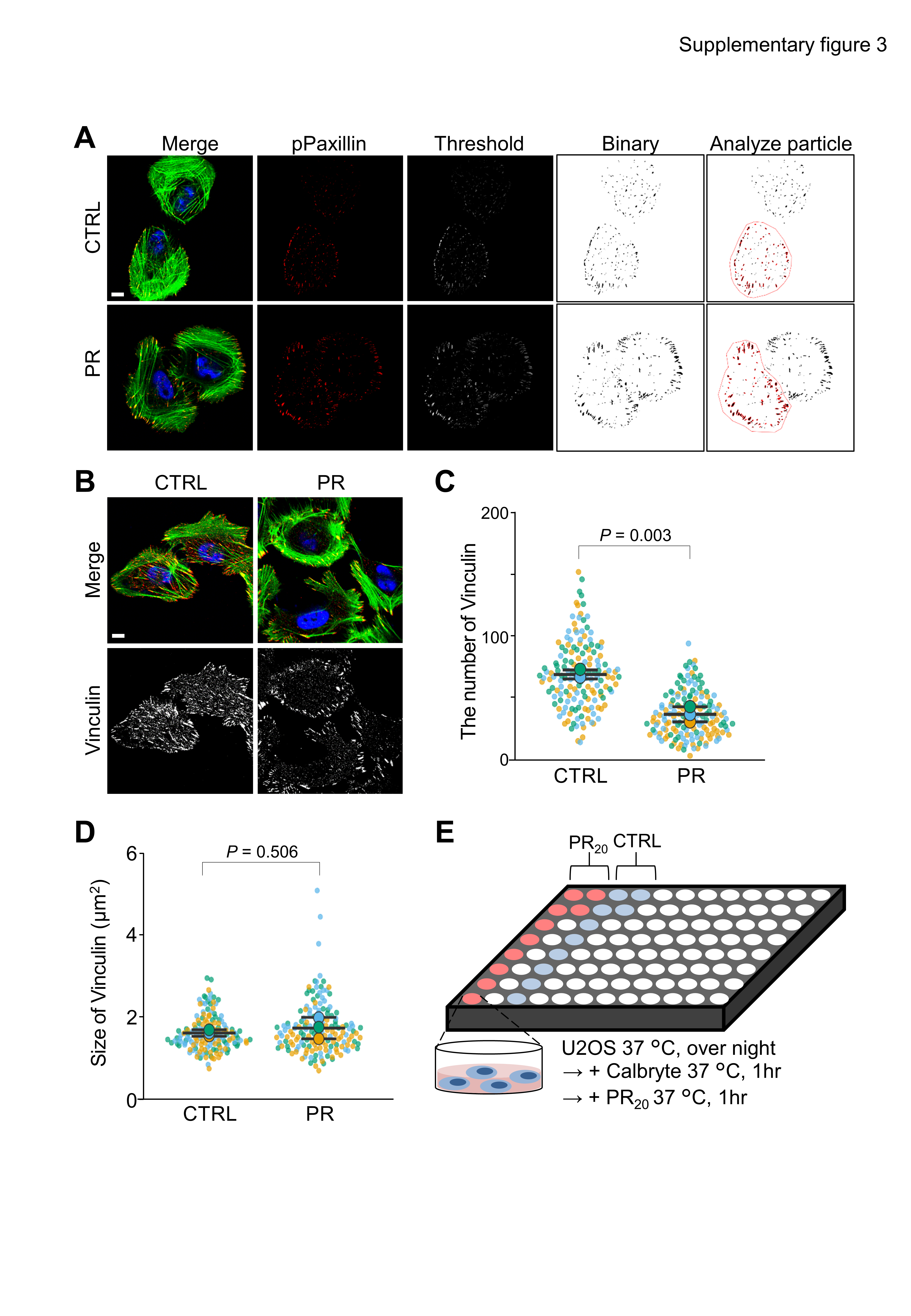

Supplement: Supplementary file 1 [file Image3.TIF]

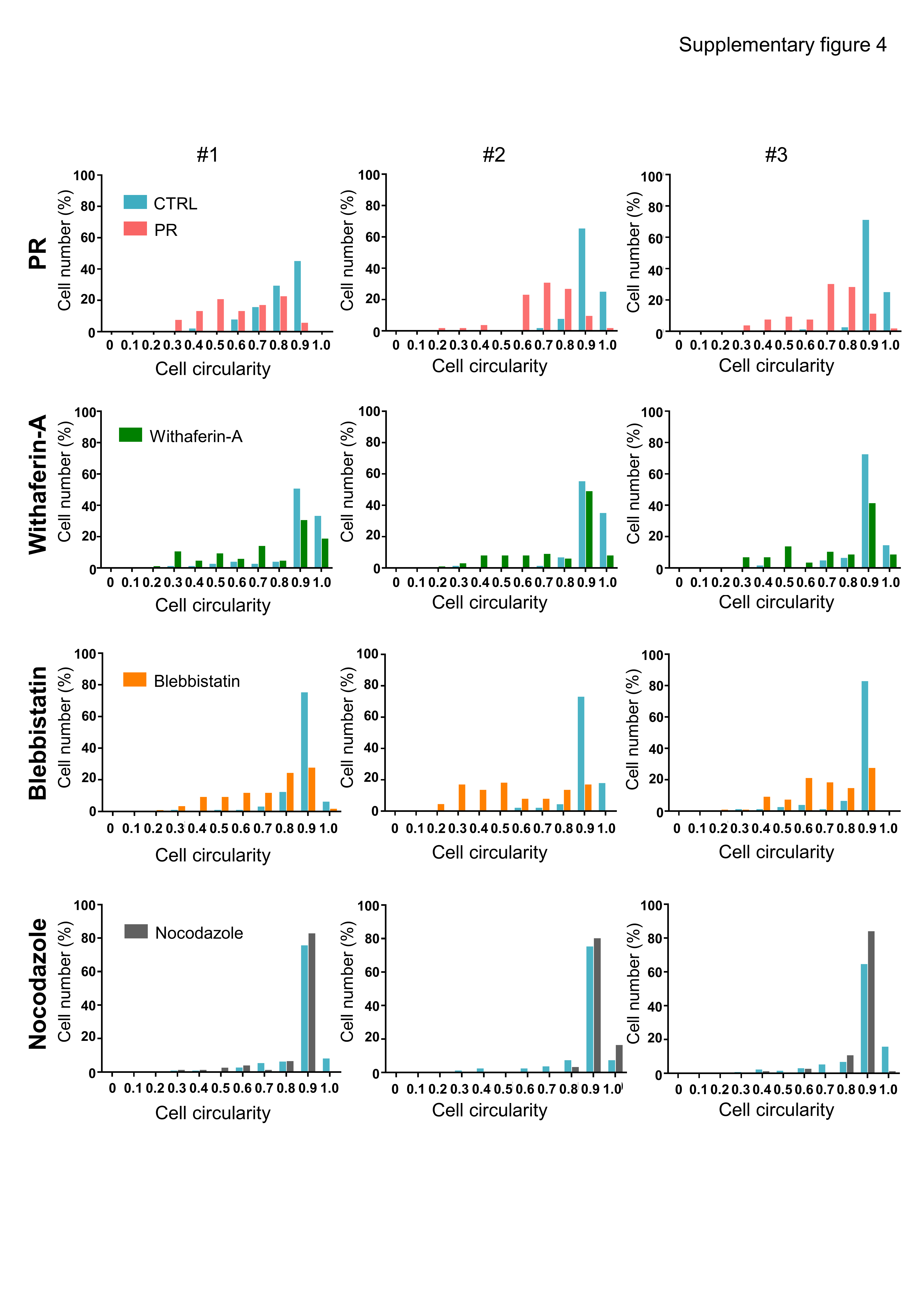

Supplement: Supplementary file 2 [file Image4.TIF]

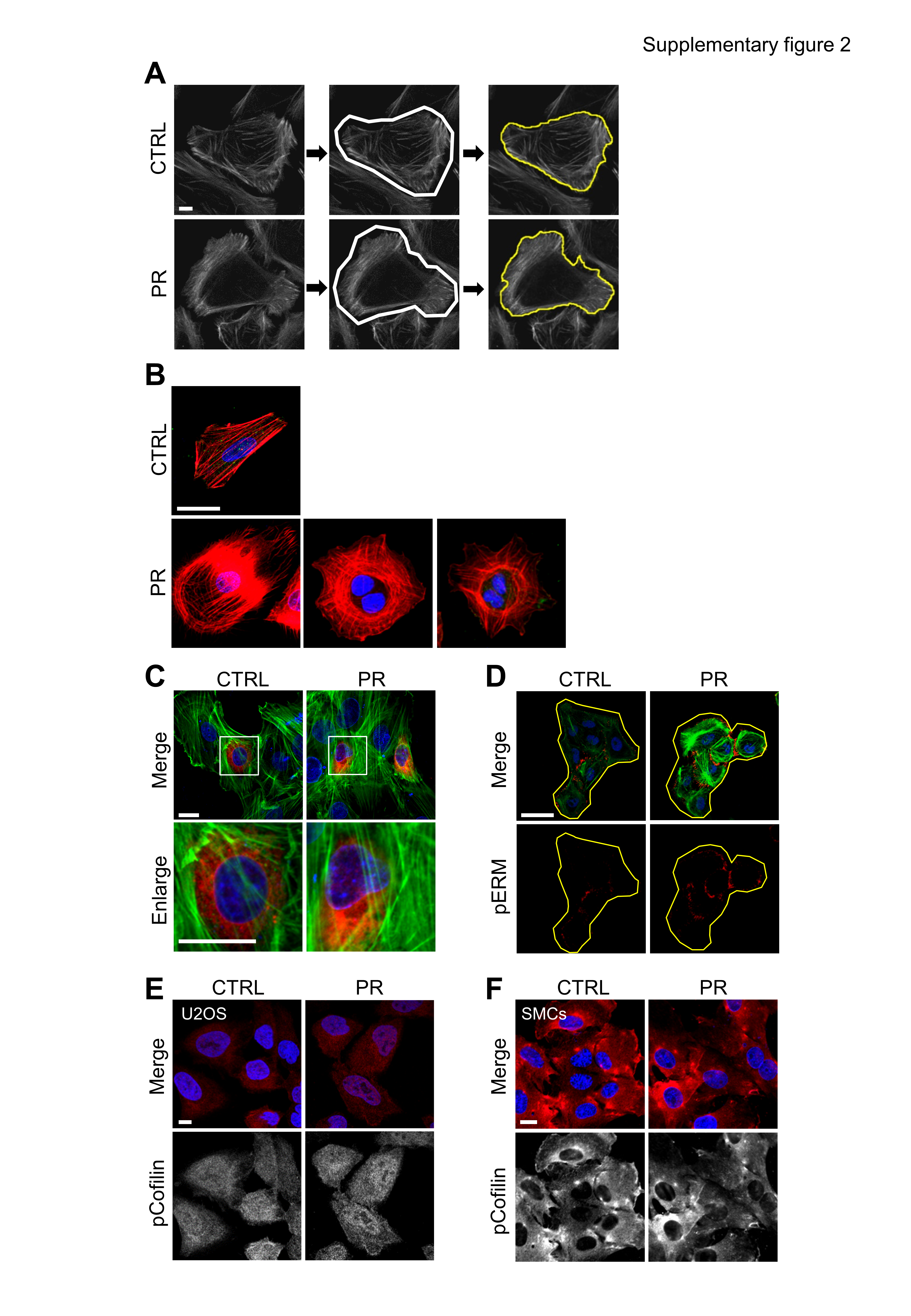

Supplement: Supplementary file 3 [file Image2.TIF]

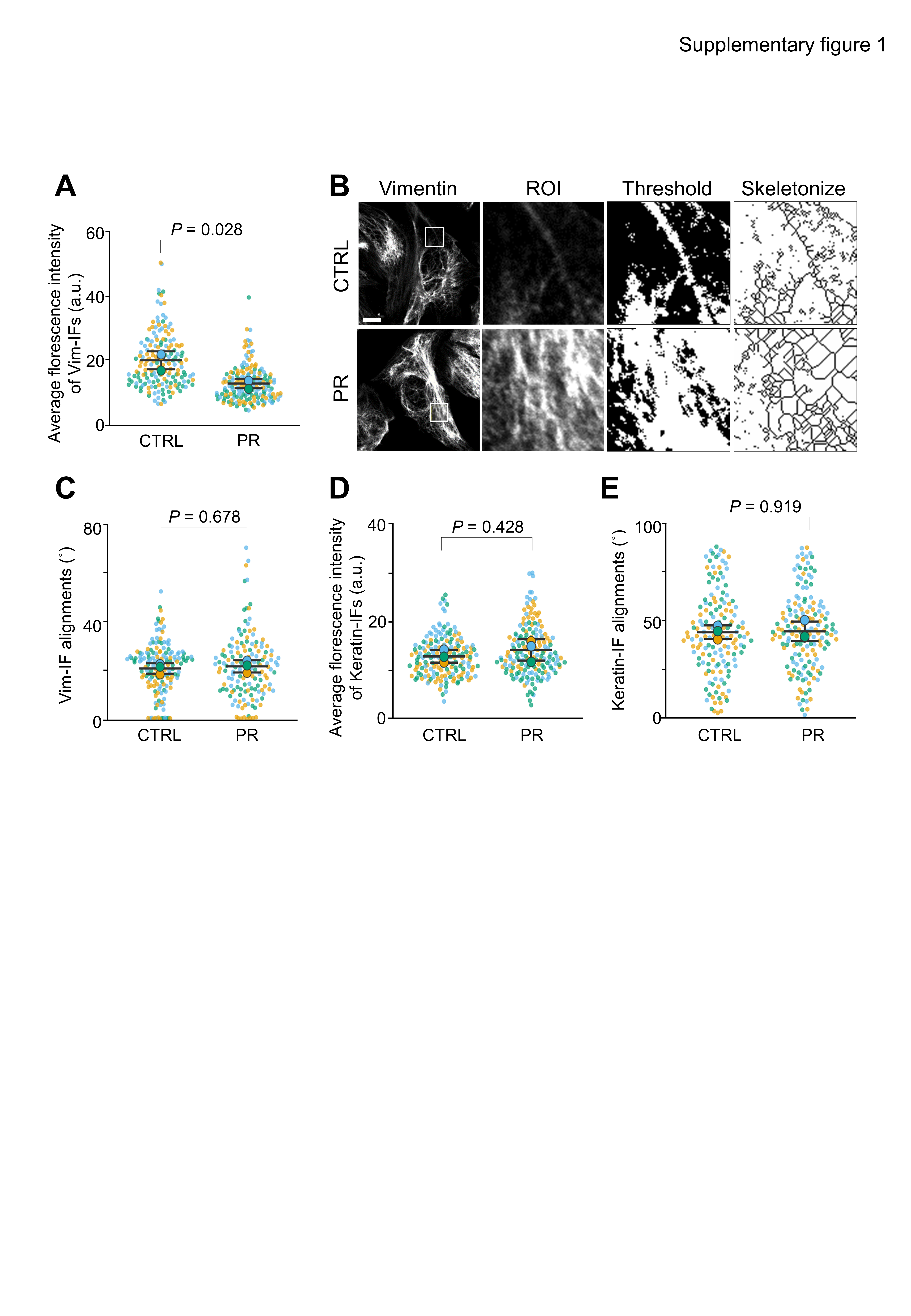

Supplement: Supplementary file 4 [file Image1.TIF]

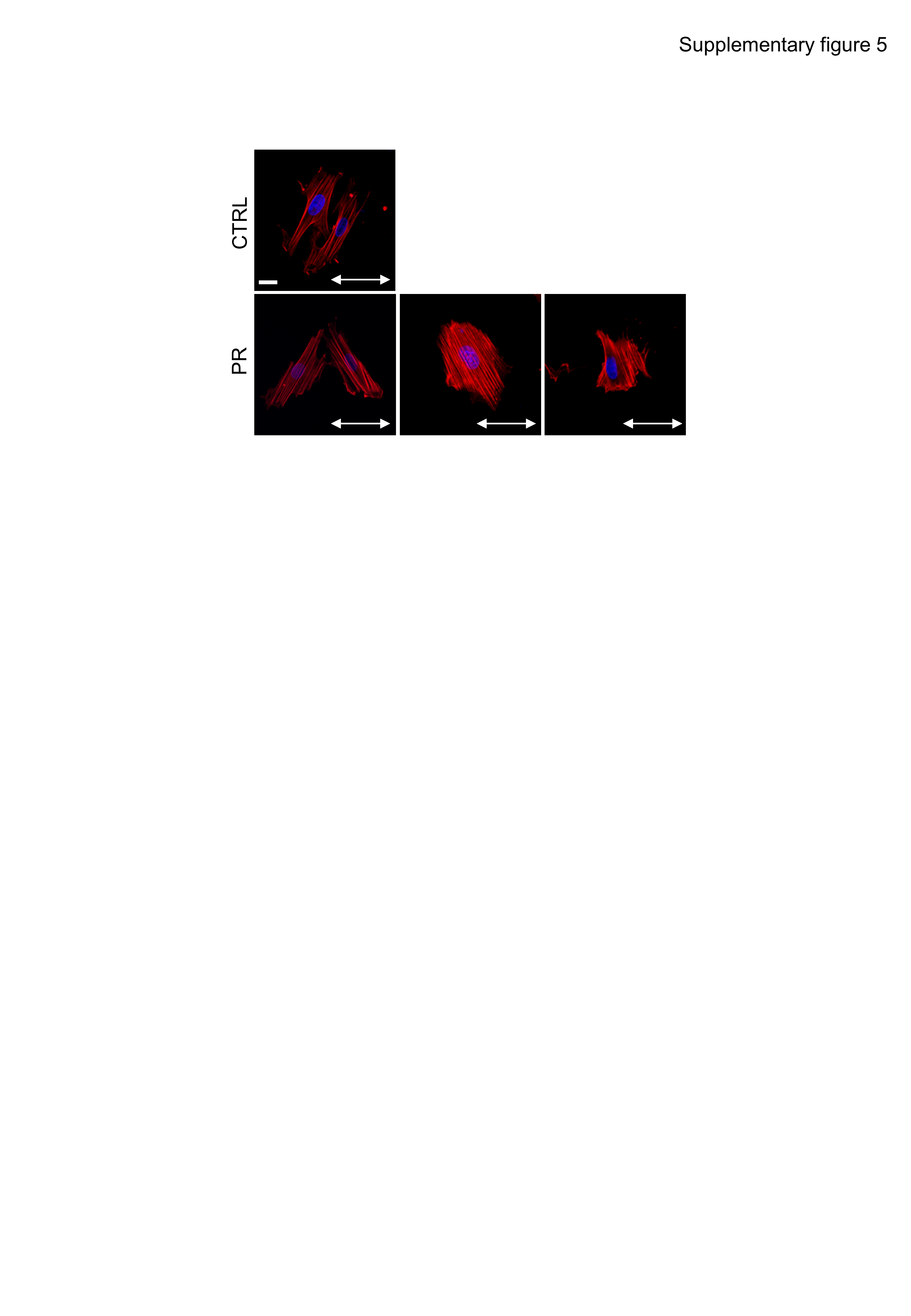

Supplement: Supplementary file 5 [file Image5.TIF]
